# Paraneoplastic Central and Peripheral Demyelination Secondary to Oesophagus Malignancy: A Case Report

**DOI:** 10.7759/cureus.93952

**Published:** 2025-10-06

**Authors:** Farheen S Bhanu, Rithvik Ramesh, Lakshmi Narasimhan Ranganathan, Sundar Shanmugam, Philo Hazeena

**Affiliations:** 1 Neurology, Sri Ramachandra Institute of Higher Education and Research, Chennai, IND

**Keywords:** combined central and peripheral demyelination, immunotherapy resistance, neurologic prognosis, oesophageal cancer, paraneoplastic neurological syndromes

## Abstract

A man in his 60s presented with subacute weakness of his lower limbs, numbness, and urinary retention, preceded by fever a few weeks back. Nerve conduction studies showed demyelinating parameters, and magnetic resonance imaging showed long-segment cord T2/STIR hyperintensities and multifocal lesions in the brain. CSF showed albuminocytologic dissociation. Despite immunotherapy with intravenous immunoglobulin (IVIg), symptoms progressed. A PET-CT scan revealed thoracic oesophageal malignancy with lymphatic spread, confirmed on biopsy as an undifferentiated carcinoma. The case was diagnosed as paraneoplastic demyelination involving both the central and peripheral nervous systems. The patient later developed pulmonary thromboembolism. This case highlights simultaneous central and peripheral demyelination as a paraneoplastic manifestation and underscores the need for malignancy screening in demyelinating syndromes unresponsive to standard immunotherapies.

## Introduction

Paraneoplastic syndromes presenting with central and peripheral demyelination have been increasingly recognised in association with select malignancies like germ cell tumours (seminomas, ovarian teratomas), plasma cell proliferative disorders, small cell carcinomas of the lung, and certain lymphomas. Recent studies linked anti-CV2/CRMP5 (anti-collapsing response-mediator protein-5) and anti-NF-186 antibodies as potential immunological markers for paraneoplastic syndromes with central and peripheral demyelination [1]. 

Oesophageal malignancies, though less commonly associated with paraneoplastic demyelinating disorders, have classically been linked with paraneoplastic cerebellar degeneration, sensory-motor neuropathies, and limbic encephalopathies [2]. Reports describing concurrent central and peripheral nervous system involvement in association with oesophageal cancer are rare. In this report, we describe a case of a man in his 60s who presented with a subacute onset of neurological symptoms consistent with combined central and peripheral demyelination, with detailed evaluation leading to the unexpected diagnosis of an underlying oesophageal carcinoma, thereby expanding the known spectrum of paraneoplastic neurological manifestations associated with oesophageal malignancy.

## Case presentation

A male patient in his 60s with known hypertension presented with weakness and numbness of his lower limbs for the last two weeks. He had noted difficulty in climbing stairs two weeks back, which progressively worsened over the next 10 days to involve difficulty in gripping his slippers as well. Four days before his presentation, the patient developed sudden worsening of his weakness, which was associated with severe low back pain. This was associated with numbness below the hip, more on the left leg than the right. A day prior to admission, he developed urinary retention. The patient had a history of low-grade fever and malaise approximately three weeks earlier.

On admission, the patient was conscious, oriented, with asymmetric bilateral lower limb proximal predominant weakness, with sparing of the upper limbs. He also had decreased sensations in his lower limbs and urinary retention. His symptoms rapidly progressed over the next two days, causing weakness of the distal lower limb as well as weakness and decreased sensations of the proximal followed by the distal upper limbs.

Investigations

**Figure 1:**
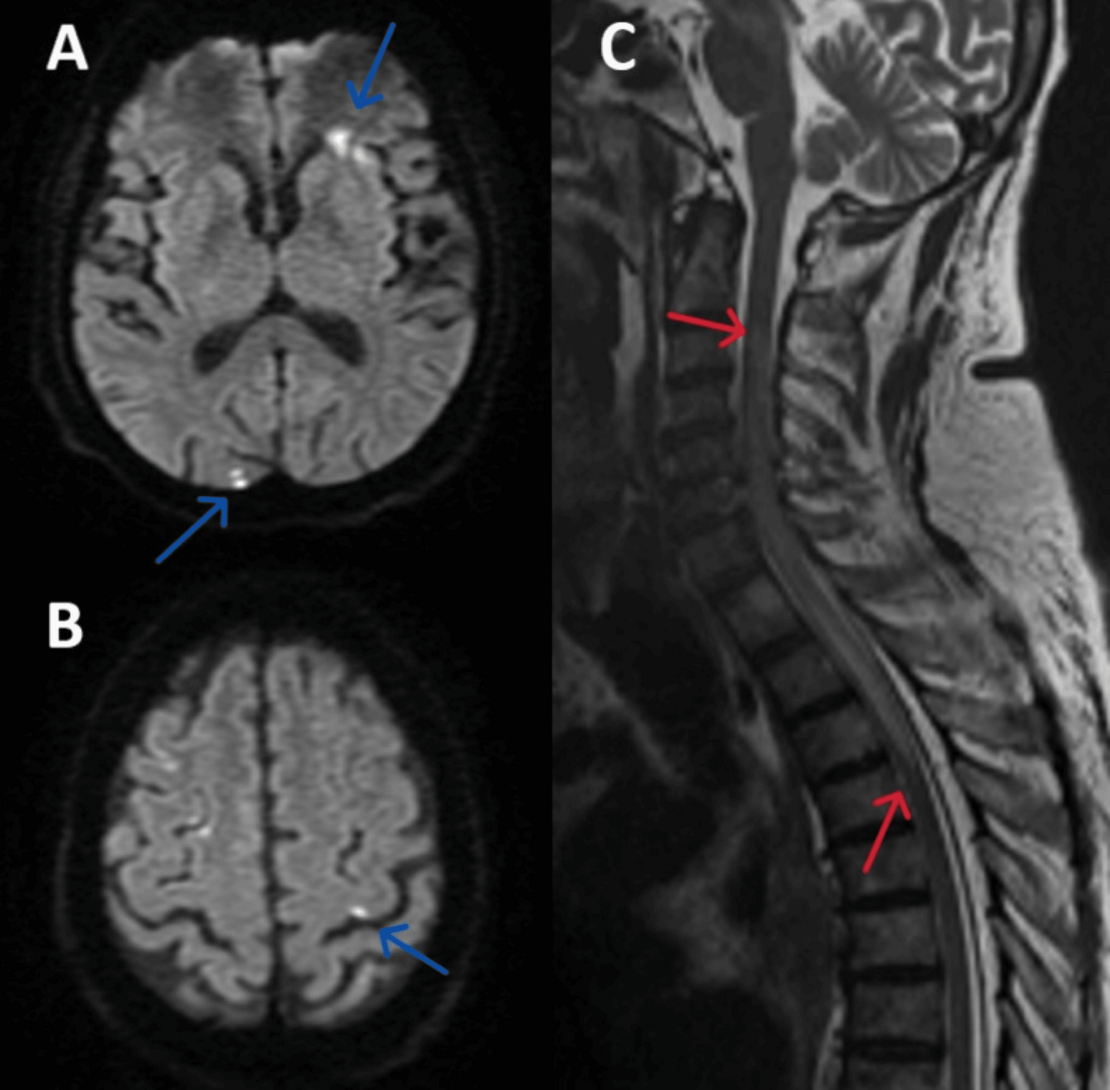
MRI findings (A, B) Areas of diffusion restrictions in bilateral cerebral hemispheres (blue arrows), (C) Long segment T2 hyperintensity in spinal cord from C3 to D3 (red arrows).

His initial workup with routine blood investigations, including hemogram, renal, liver, glucose, and thyroid profiles, was normal. MRI of the dorso-lumbar spine revealed long-segment T2 and short tau inversion recovery (STIR) hyperintensity (C3 to D3) with subtle diffusion restriction, raising suspicion of acute demyelination or infarction (Figure 1). Nerve conduction study showed demyelinating motor radiculoneuropathy of bilateral lower and upper limbs. CSF analysis was done, which showed no cells, elevated protein (83.3 mg/dL; reference range: 15-45 mg/dL). Tests for infections, including viral panels, tuberculosis, and bacterial etiology, were negative. Serum calcium and angiotensin-converting enzyme (ACE) levels were normal. Due to a transient drop in sensorium, an MRI brain was performed, which showed multiple areas of diffusion restriction and fluid-attenuated inversion recovery (FLAIR) hyperintensity in bilateral cerebral hemispheres, including the left frontoparietal region, subcortical white matter, and insular cortex (Figure 1), suggestive of acute demyelination or embolic infarctions, thereby increasing diagnostic complexity.

Differential diagnosis

This elderly male patient presented with an initial, gradually progressive weakness of his lower limbs for a two-week duration, with a sudden worsening associated with additional sensory involvement and bladder symptoms. This would suggest either a myeloradiculoneuropathy (with the spine component following the radiculoneuropathy) or an expansile spinal lesion (with sudden worsening) as the site of the lesion. The examination at presentation did not help differentiate between upper motor neuron vs lower motor neuron involvement, as the duration was subacute. Nerve conduction studies revealed demyelinating features, while spinal MRI showed a long-segment hyperintensity-findings consistent with central demyelination. These collectively pointed towards an immune-mediated demyelinating process.

**Figure 2:**
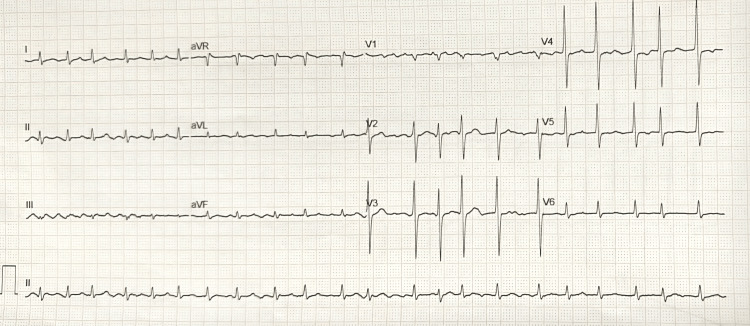
ECG (12 lead) of the patient showing atrial fibrillation.

But the in-hospital worsening, which led to brain imaging showing lesions, brought into consideration the possibility of ischemia being the etiology behind the lesions in the spine and the brain. This was supported by the presence of atrial fibrillation (Figure 2), which could cause embolic showers. The possibility of both demyelinating and ischemic features brought into consideration the diagnostic philosophies of Occam's razor versus Hickam’s dictum. Occam’s razor would suggest a single unifying diagnosis, immune mediated lesions, as the explanation for both the central and peripheral findings. This was supported by the subacute progression, preceding infection, objective demyelination in the nerve conduction studies (NCS), and the albuminocytological dissociation in CSF. While this remains theoretically possible, the distinct radiological pattern of the brain lesions (peripheral more than central), their acute onset, and the documented atrial fibrillation point toward a separate cardioembolic mechanism. Therefore, Hickam’s dictum, which allows for the coexistence of multiple pathologies, appears more appropriate in this case. It is more likely that the patient had two concurrent disease processes: a demyelinating syndrome affecting the spinal cord and peripheral nerves, and embolic cerebral infarctions secondary to atrial fibrillation. This dual pathology better accounts for the heterogeneity of clinical and imaging findings and explains the poor response to immunotherapy.

Treatment

**Figure 3:**
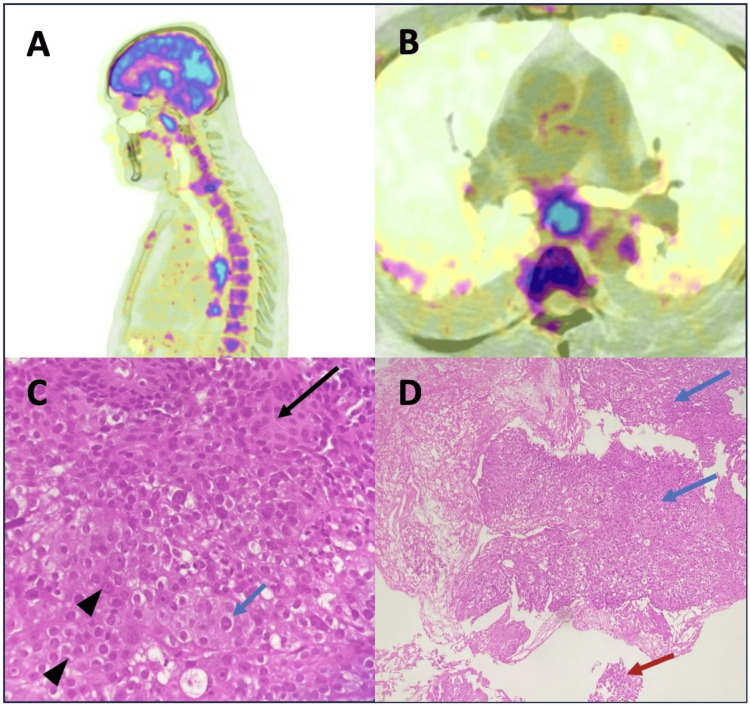
PET/CT and biopsy findings (A, B) PET/CT whole body showing malignant growth in the thoracic oesophagus; (C) Pleomorphic cells with abundant eosinophilic cytoplasm (black arrow heads), with large vesicular nuclei and prominent nucleoli (blue arrow), with individual cell keratinisation (black arrow); (D) Infiltrating tumor composed of atypical cells arranged in sheets and nests (blue arrow), with high grade dysplasia in the adjacent epithelium (red arrow).

Considering a possible ongoing autoimmune condition, the patient was given five cycles of IVIg, following which there was no improvement in symptoms. Anticoagulation and antiplatelet therapy were also started following suspicion of embolic stroke from atrial fibrillation. Considering his older age and poor response to IVIg, a paraneoplastic condition was suspected. PET/CT of the whole body was done, which showed suspicious malignant growth in the thoracic oesophagus with lymphatic spread (Figure 3A, 3B). Upper gastrointestinal (UGI) endoscopy-guided biopsy was done that showed an undifferentiated, highly malignant growth in the thoracic oesophagus (Figure 3C, 3D).

Outcome and follow-up 

In the days following diagnosis, the patient developed pulmonary thromboembolism and continued clinical decline. No neurological recovery was noted despite IVIg, and further treatment was limited due to his poor general condition.

## Discussion

This case describes a man in his 60s presenting with immune-mediated lesions in the peripheral nerve, spine, and the brain, and was eventually diagnosed to have a stage 4 oesophageal carcinoma. Paraneoplastic neurological syndromes (PNS) are immune-mediated disorders that may precede the diagnosis of cancer as early as five years [3]. Although PNS are commonly described in association with small-cell lung and other cancers, fewer than 200 cases related to oesophageal cancer have been reported in the literature to date, spanning across all the histological subtypes [2]. The most frequently encountered PNS reported in association with oesophageal malignancies are limbic encephalitis, cerebellar degeneration (adenocarcinoma, sensory or sensory motor neuropathy, opsoclonus myoclonus syndromes, Lambert-Eaton myasthenic syndrome (LEMS), and necrotising myelopathy [2]. The associated characteristics and the antibody in the literature have been tabulated in Table 1 [4-10]. However, many oesophageal malignancies are seronegative, making whole-body PET scans integral in early diagnosis.

**Table 1:** Paraneoplastic syndromes in esophageal malignancy IVIg: intravenous immunoglobulin

Syndrome	Response to Immunotherapy	Associated Antibody/Antibodies
Limbic Encephalitis (LE) [4]	Partial or complete response in some cases; better with early treatment	Anti-Hu, Anti-Ma, Anti-NMDAR, Anti-GABA(B)
Paraneoplastic Cerebellar Degeneration [5]	Poor response; progressive and irreversible in all reported cases	Anti-Yo (PCA-1)
Peripheral Neuropathies / Neuronopathies [6]	Mixed outcomes; stabilization in some cases, limited improvement in others	Anti-Hu, Anti-GD1a, GD1b, GM1 (ganglioside antibodies)
Opsoclonus–Myoclonus Syndrome (OMS) [7]	Good response; rapid improvement after IVIG in both cases	None detected
Neuromyelitis Optica Spectrum Disorder (NMOSD) [8]	Marked improvement after definitive cancer therapy	Anti-Aquaporin-4 (AQP4)
Paraneoplastic Necrotizing Myelopathy [9]	Good recovery of strength with steroids	None detected (AQP4-negative)
Lambert–Eaton Myasthenic Syndrome [10]	Stabilized with cancer treatment and IVIg	P/Q-type Voltage-Gated Calcium Channel (VGCC)

Myelopathic paraneoplastic syndromes in oesophageal malignancy, though rare, represent a challenging manifestation [9]. This presents as subacute or acute myelopathies characterized by paraparesis or paraplegia, sensory deficits, bladder and bowel dysfunction, with long-segment spinal cord lesions on MRI. Among these, a subtype, paraneoplastic necrotizing myelopathy (PNM), has been reported frequently in patients with squamous cell carcinoma of the oesophagus. IN the absence of a known malignancy diagnosis, this clinical presentation can mimic demyelinating disorders like NMOSD or spinal cord infarction [9], as in our case. The prognosis is variable and depends on the underlying histology subtype. In the few reported cases of PNM with longitudinally extensive transverse myelitis secondary to esophageal cancer, outcomes have ranged from partial neurological recovery to irreversible deficits or death. For example, Urai et al. reported a case of PNM associated with squamous cell carcinoma where high-dose corticosteroids led to significant improvement in motor strength, though the patient ultimately succumbed to the malignancy several months later due to progressive systemic disease [9]. Ischemic lesions are also very common in malignancy patients, arising due to both tumor-related and systemic factors, and are a close differential to the imaging lesions [11].

While PNS are well recognised in many solid tumors, their relationship with esophageal malignancies remains unexplored and poorly characterised. Given the rising incidence of esophageal cancer and the potential for serious and a vast spectrum of neurological complications, it is important to broaden clinical awareness and research into these associations.

## Conclusions

PNS may involve both the central and peripheral nervous systems and can mimic demyelinating or ischemic syndromes. Multifocal CNS lesions in older adults should raise suspicion for cardioembolic stroke, especially with atrial fibrillation. Inadequate response to treatment or lack of response to immunotherapy in demyelinating presentations should prompt evaluation for occult malignancies. PET-CT and autoimmune panels are critical in evaluating atypical demyelinating presentations. Paraneoplastic demyelination secondary to esophageal carcinoma, though rare, should be considered in rapidly progressive, treatment-refractory cases.
